# Energy Consumption Model for Sensor Nodes Based on LoRa and LoRaWAN

**DOI:** 10.3390/s18072104

**Published:** 2018-06-30

**Authors:** Taoufik Bouguera, Jean-François Diouris, Jean-Jacques Chaillout, Randa Jaouadi, Guillaume Andrieux

**Affiliations:** 1 University of Bretagne Loire, Polytech Nantes, IETR, 44300 Nantes, France; jean-francois.diouris@univ-nantes.fr (J.-F.D.); guillaume.andrieux@univ-nantes.fr (G.A.); 2 CEA Leti/Tech, MINATEC Campus, 38054 Grenoble, France; jean-jacques.chaillout@cea.fr; 3 University of Angers, IUT Angers, LARIS, 49035 Angers, France; randa.jaouadi@univ-angers.fr

**Keywords:** communicating sensors, energy consumption, autonomy constraint, LoRa, LoRaWAN, energy optimization

## Abstract

Energy efficiency is the key requirement to maximize sensor node lifetime. Sensor nodes are typically powered by a battery source that has finite lifetime. Most Internet of Thing (IoT) applications require sensor nodes to operate reliably for an extended period of time. To design an autonomous sensor node, it is important to model its energy consumption for different tasks. Each task consumes a power consumption amount for a period of time. To optimize the consumed energy of the sensor node and have long communication range, Low Power Wide Area Network technology is considered. This paper describes an energy consumption model based on LoRa and LoRaWAN, which allows estimating the consumed power of each sensor node element. The definition of the different node units is first introduced. Then, a full energy model for communicating sensors is proposed. This model can be used to compare different LoRaWAN modes to find the best sensor node design to achieve its energy autonomy.

## 1. Introduction

Wireless Sensor nodes enable a wide range of applications, such as infrastructure security, environment monitoring, event detection, etc. [1]. Such applications have the purpose of collecting information about a given phenomena or event. These sensor nodes are generally deployed in harsh and inaccessible environments. For these reasons, sensor nodes are supposed to operate over long time periods without human intervention [1,2].

Modeling of energy consumption is an important consideration in the design of communicating sensors for monitoring a specific target application. Communicating sensors should perform the following tasks for most applications [1,3]: sense events, local information processing of sensed events and packet transmission to the access point [3,4]. Each task consumes a power consumption amount during a given time duration. Therefore, an accurate energy consumption model of the sensor node is essential to estimate the sensor lifetime [5]. This energy model allows optimizing the power consumption of the sensor node.

The proposed energy model is based on LoRa and LoRaWAN technologies. The LoRa system operates at the *ISM* frequency bands, which makes it an attractive solution for the Internet of Things and Machine to Machine (*M2M*) systems [6,7]. Its low power associated to the long range communication pushes LoRa to the top of the LPWAN technologies. Due to its unique modulation, LoRa is a very versatile technology that can be adapted to different environment types and application classes [8].

In this paper, we focus on the LoRaWAN protocol which was developed by LoRa Alliance to serve IoT applications. The LoRaWAN characteristics have a great influence in determining the battery lifetime of the sensor node [9,10]. In fact, using LoRaWAN technology can optimize the energy consumption by adapting its principal parameters.

The goal of this work is to propose an energy consumption model for sensor nodes using LoRa modulation and LoRaWAN protocol. This model is evaluated using different LoRaWAN modes. Besides, we study the impact of LoRaWAN parameters such as acknowledged transmission, spreading factor, coding rate, payload size and communication range on the sensor node consumption.

The rest of the paper is organized as follows. Section 2 presents related works. The wireless sensor design is described in Section 3. Section 4 depicts our energy consumption model. The LoRa modulation technique and the LoRaWAN characteristics are exposed in Section 5. Numerical results are explained in Section 6. Finally, conclusion and future works are given in Section 7.

## 2. Related Works and Contributions

LoRa and LoRaWAN technologies are relatively recent standards [11]. Most existing research based on LoRa and LoRaWAN has focused on features such as delay, range, throughput and network capacity [8,11,12,13]. Since the LoRa modulation is deployed for sensor applications, several papers evaluated this new technology with respect to its energy consumption.

Driven by the challenges of energy consumption of wireless sensor applications, many recent works have focused on the power dissipation of communicating sensors. Terrasson, et al. present an energy model for Ultra-Low Power sensor nodes in [14,15]. In these papers, the authors described the modeling of a sensor node dedicated to wireless sensor network applications. However, the RF module used in this study was the *CC1100* module (a short range device) which did not include the LoRa technology. An other energy estimation model is presented in [16], The goal of this work is to obtain a low power consumption of sensor nodes. To save power, Mare S. et al. have concluded that the communication module and the microcontroller must be in idle state as long as possible when they are not active. This work proposes interesting results, but LoRa and LoRaWAN technologies are not integrated into this study. Phui, et al. proposed a comparison of LoRaWAN classes and their power consumption in [17]. The objective of this study is to offer an experimental comparison of LoRaWAN classes to verify the published current levels of different operating modes in LoRa datasheets. The measurement results allow to estimate the lifetime of end-node devices. However, in this paper, the authors did not study the effect of different LoRaWAN parameters such as coding rate, communication range and transmission power level on the total consumed energy.

Many other studies provide the power consumption of sensor nodes based on LoRa/LoRaWAN. Most of the current values were obtained from the datasheet or by empirical means [18,19,20], without developing an energy model that can estimate and optimize the energy consumption of the wireless sensors. Casals, et al. developed current models that allow the characterization of LoRaWAN devices lifetime and energy cost in [21]. The proposed models were very important and derived from measurement results using a currently prevalent LoRaWAN platform. However, Casals, et al. did not include the energy consumption of the processing and the sensor units. In our paper, we have modeled these units for an application scenario of connected sensor. Another major difference is that we have illustrated our energy model with optimization of LoRaWAN parameters such as the spreading factor *SF*, the coding rate *CR*, the bandwidth *BW*, the payload size and the communication range. Optimizing these parameters is very important to reduce the energy consumption of the sensor node.

The previous works were proposed to estimate the amount of energy consumption by a sensor node. Some of these studies have not included LoRa technology into their energy models, so they used different RF transceivers which are mainly dedicated to short range communication. Other works did not study the energy optimization of sensor nodes. In fact, optimizing LoRa and LoRaWAN parameters are very interesting to reduce the energy consumption by the communicating sensor.

To estimate and optimize the consumed energy by the sensor node, we propose a full energy model based on LoRa/LoRaWAN technologies. This model is based on LoRaWAN Class A, which is the most energy efficient class in the protocol. The proposed model takes into account the modeling of the sensor node units and especially the processing and the sensor units using a real IoT application. Then, different LoRaWAN transmission modes are studied to choose the best mode that can optimize energy consumption. Besides, a detailed optimization study of LoRa/LoRaWAN parameters such as spreading factor, coding rate, bandwidth, communication range and transmission power is presented to maximize the sensor lifetime. The energy model takes into account the transmission acknowledgment and its energy consumption cost using different LoRaWAN scenarios.

## 3. Sensor Node Design

**Figure 1:**
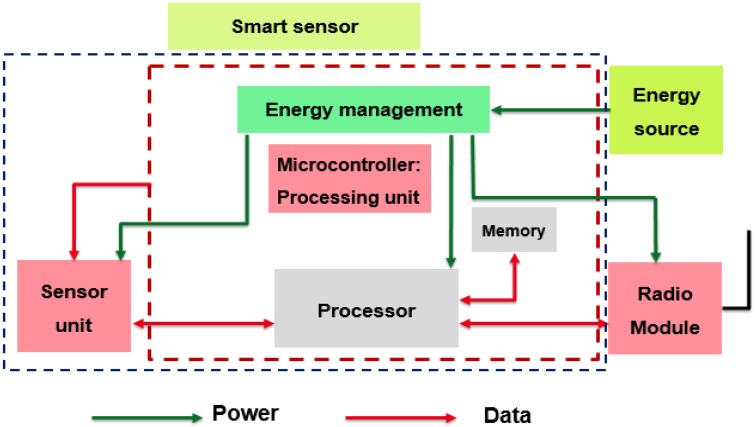
Sensor node architecture.

The proposed node model is depicted in Figure 1. The sensor node can transmit data to an access point using the LoRa/LoRaWAN radio module. To carry out its different operations, the sensor needs an embedded energy source [22]. This source is a battery in this study. In this paper, we consider a connected sensor to measure the acceleration values. Internal memory integrated into the microcontroller is enough for this type of application and we don’t consider external memory.

The three main units of the sensor are the sensor unit, the processing unit and the radio module. Each block is briefly described in the following paragraphs.

### 3.1. Sensor Unit

The sensor unit can detect and respond to different inputs from the environment. These specific inputs can be light, heat, pressure, acceleration, etc. The input signal is generally an analog signal that is converted to digital form with an Analog to Digital Converter (ADC). The ADC unit is integrated into the sensor unit in this study. Both sensing and conversion processes take time and consume power [11].

### 3.2. Processing Unit

The processing manages all the resources utilized for the system operation [22]. This block is responsible for acquisition of output signal from the sensor unit, process the data after acquisition and communicate these data to the LoRa transceiver. Our embedded system is based on the *STM32L073* microcontroller from ST Microelectronics [12]. This microcontroller can be optimized for very low power consumption.

### 3.3. Communicating Unit

Once the essential data processing is done, the information can be communicated to the access point [22]. Several standards have been proposed in the recent years for the IoT applications [11,12]. Among these, Low-Power Long-Range radio (LoRa and LoRaWAN) are gaining a lot of interest because of the high receiver sensitivity (down to −137 dBm). These technologies can reach long transmission distance (up to 20 km). In our design model, LoRa/LoRaWAN are implemented using the Semtech’s *SX1272* transceiver.

## 4. Energy Consumption Model

To study the node autonomy, it is necessary to model each node block. In this section, we present different operating modes of sensor nodes. Then, the consumed energy of each mode is calculated and the consumption energy model is deduced.

### 4.1. Methodology and Assumptions

**Figure 2:**
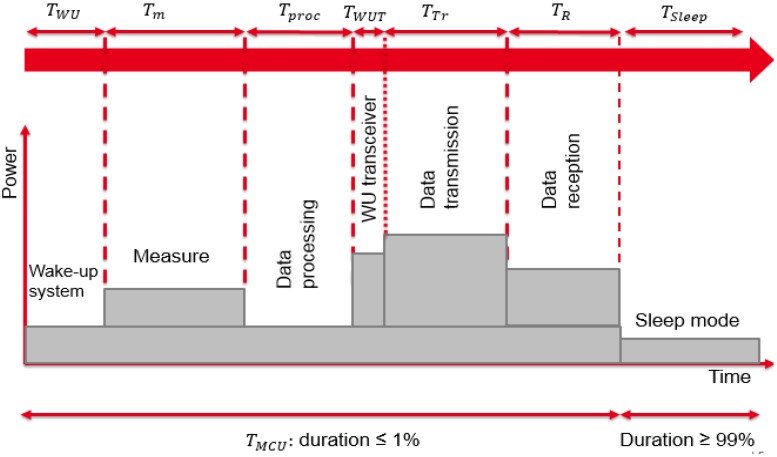
Sensor working scenario.

Figure 2 illustrates a possible working sequence of the sensor node and allows defining different operating modes, which are managed by the processing unit.

Our energy consumption model is based on the following assumptions:

- As proposed in [1,4,14,15], the processing unit is in on-state along the working sequence. To optimize the consumed power by the MCU unit, this assumption can be improved to make the MCU unit in low-power modes during most of the activity cycle.
- Each step of the sensor working sequence is characterized by a constant time duration.
- The radio module transmits a packet of information at a fixed transmission power level.
- No local storage of information is considered in this model but we assume a real-time transmission of all measured data.

### 4.2. Proposed Energy Model

This part defines the different operating modes of the communicating sensor. A first approach consists in considering all elements active during a fixed time duration and inactive for the rest of the time cycle. Most of the time, the wireless sensor is in sleep mode. The consumed energy in this mode could impact the power consumption amount of the sensor. Then, it is necessary to consider the sleep mode consumption in our model. In this study, all peripherals are powered at the same voltage level equal to 3.3 V, except the sensor unit which is powered at 2 V. The total consumed energy $E_{Total}$ used by the communicating sensor for one cycle is given by Equation (1):

(1) $$E_{Total}=E_{Sleep}+E_{Active},$$

where $E_{Sleep}$ and $E_{Active}$ are the dissipated energy by the node in the sleep mode and the total energy consumption during the active mode of the microcontroller, respectively. $E_{Sleep}$ is expressed as:

(2) $$E_{Sleep}=P_{Sleep}\cdot T_{Sleep},$$

where $P_{Sleep}$ and $T_{Sleep}$ are the power consumption and the time duration in the sleep mode, respectively.

The total energy consumption $E_{Active}$ is calculated as the sum of the energy consumption of each part of the sensor node. It is given by the following equation:

(3) $$E_{Active}=E_{WU}+E_{m}+E_{proc}+E_{WUT}+E_{Tr}+E_{R},$$

where $E_{WU}$, $E_{m}$, $E_{proc}$, $E_{WUT}$, $E_{Tr}$ and $E_{R}$ are, respectively, the consumed energies in the system wake-up, the data measurement, the microcontroller processing, the wake-up of the LoRa transceiver, the transmission mode and the reception mode. Then, before doing measurements, the wake-up of the sensor node is managed by the processing unit. The consumed energy $E_{WU}$ during the wake-up duration $T_{WU}$ is given by:

(4) $$E_{WU}=P_{ON}\left(f_{MCU}\right)\cdot T_{WU},$$

where $P_{ON}\left(f_{MCU}\right)$ and $T_{WU}$ are the consumed power by the microcontroller (which depends on the microcontroller frequency $f_{MCU}$) and the wake-up duration, respectively.

After the wake-up time, the sensor realizes data measurements. Equation (5) presents the amount of dissipated energy $E_{m}$ during this phase:

(5) $$E_{m}=\left(P_{ON}\left(f_{MCU}\right)+P_{m}\right)\cdot T_{m},$$

where $P_{m}$ and $T_{m}$ are the consumed power and the corresponding time duration of measurements, respectively.

After the measurement step, the microcontroller proceeds to data processing. The time duration $T_{proc}\left(f_{MCU}\right)$ depends on microcontroller operating frequency $f_{MCU}$ and on the number of instructions $N_{inst}$ ($T_{proc}\left(f_{MCU}\right)=\frac{N_{inst}}{f_{MCU}}$). In Equation (6), we calculate the consumed energy by the processing unit (we assume one instruction/clock period):

(6) $$E_{proc}=P_{ON}\left(f_{MCU}\right)\cdot T_{proc}\left(f_{MCU}\right)$$

Then, the consumed energy $E_{WUT}$ during the transceiver wake-up $T_{WUT}$ is given by:

(7) $$E_{WUT}=\left(P_{ON}\left(f_{MCU}\right)+P_{WUT}\right)\cdot T_{WUT},$$

where $P_{WUT}$ is the consumed power during the wake-up of the transceiver. After that, the consumed energy $E_{Tr}$ by the transmit mode is expressed as:

(8) $$E_{Tr}=\left(P_{ON}\left(f_{MCU}\right)+P_{Tr}\right)\cdot T_{Tr},$$

where $P_{Tr}$ is the dissipated power by the transmit mode and $T_{Tr}$ is its time duration, it is given by Equation (9):

(9) $$T_{Tr}=N_{bit}\cdot T_{bit},$$

where $N_{bit}$ and $T_{bit}$ are, respectively, the number of transmitted bits and the duration of one bit transmission.

In the case of acknowledgment transmission, the consumed power by the sensor node denoted $E_{R}$ is given by the following equation:

(10) $$E_{R}=\left(P_{ON}\left(f_{MCU}\right)+P_{R}\right)\cdot T_{R},$$

where $P_{R}$ is the dissipated power by the reception mode and $T_{R}$ is the corresponding time duration.

Moreover, the consumed energy by the microcontroller $E_{MCU}$ in on-state is given in Equation (11):

(11) $$E_{MCU}=P_{ON}\left(f_{MCU}\right)\cdot T_{MCU}\left(f_{MCU}\right),$$

where the microcontroller time duration $T_{MCU}\left(f_{MCU}\right)$ depends on the overall working time in the different modes. It can be written as the following:

(12) $$T_{MCU}\left(f_{MCU}\right)=T_{WU}+T_{m}+T_{proc}\left(f_{MCU}\right)+T_{WUT}+T_{Tr}+T_{R},$$

After developing our energy model, the next section depicts the characteristic of the communication link between the sensor node and the gateway, using the LoRa/LoRaWAN technologies.

## 5. LoRa and LoRaWAN Technologies

In this section, we discuss several LoRa/LoRaWAN characteristics. We start by introducing the overview of these technologies. Then, the different LoRa/LoRaWAN basics are presented.

### 5.1. LoRa and LoRaWAN Overview

The LoRa is a long range technology with low power consumption that uses the ISM band (the unlicensed radio spectrum in the industrial, scientific and medical radio band). The objectives of this technology is to increase sensor battery lifetime and reduce the device cost [23]. LoRa uses the CSS modulation (Chirp Spread Spectrum) to maintain low power characteristics for the benefit of increasing communication range [9]. The LoRaWAN is a wireless communication protocol developed by LoRa Alliance to serve for different challenges faced with long range communication in IoT applications [9].

**Figure 3:**
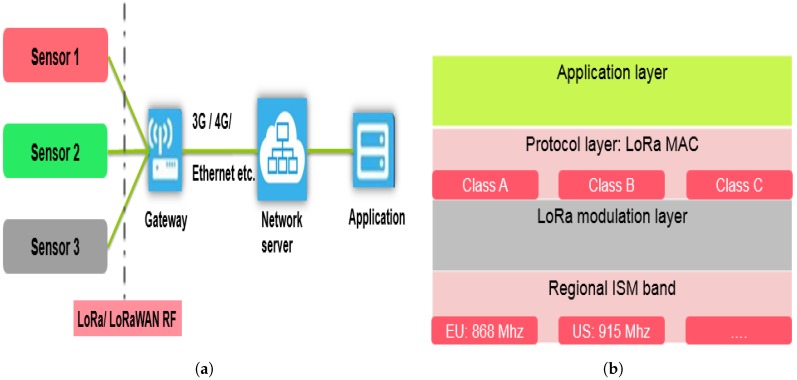
(**a**) LoRaWAN network architecture; and (**b**) LoRaWAN protocol stack.

Figure 3a depicts the LoRaWAN network architecture. The End-devices (different sensor types) communicate to the gateway using LoRa/LoRaWAN RF interface. The gateway transmits frames to the server through a non-LoRaWAN network such as Ethernet, 3G/4G, Wi-Fi, etc. [24]. Figure 3b shows the LoRaWAN communication stack. As can be seen, the physical layer defines the ISM band (868 Mhz in Europe). In this paper, we focus on LoRa modulation without considering the Frequency Shift Keying (FSK) modulation because the LoRa modulation is more adapted to long range communication with low power consumption as indicated in [25]. The CSS modulation has been implemented by Semtech in the LoRa modulation layer. Then, the LoRa Alliance has defined the LoRaWAN communication protocol specifications in the protocol layer [26].

**Figure 4:**
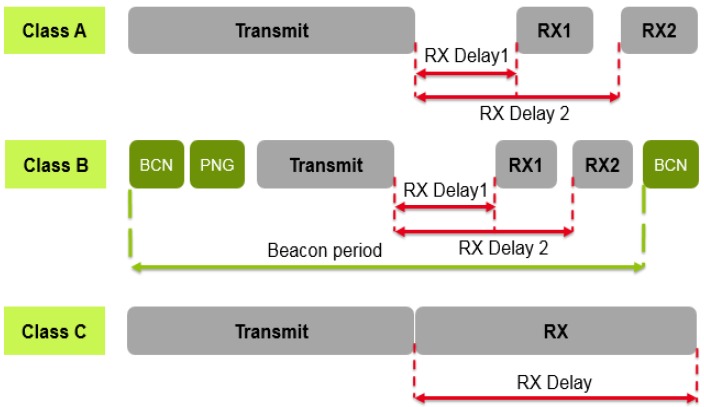
Different LoRaWAN classes.

Taking into account the application needs, The LoRaWAN specification defines three classes available for different power usage strategies [26]. These classes are illustrated in Figure 4 and can be briefly described as follows:

1. Class A: In this case, sensor can initiate an uplink transmission based on their own needs. This class allows bi-directional communication, each uplink transmission is followed by two short downlink messages. Class A has the lowest power consumption [26,27].
2. Class B: The gateway initiates the communication by transmitting downlink messages (ping slots), so the end-device can receive additional windows at scheduled fixed time-intervals. A periodic beacon from the gateway is required for synchronization. This class has the medium power consumption [27,28].
3. Class C: End-devices of this class have nearly continuously open receive windows, which can only be closed when transmitting. Class C end-devices use more power to operate than Class A or B but they offer the lowest latency for server to end-device communication [27].

Table 1 summarizes the LoRaWAN class characteristics. As mentioned before, all transactions are started by the sensor in the case of Class A, whereas the network server can only transmit two downlink slots [17]. Then, the radio receiver stays active until the downlink frame is demodulated. If a frame was detected and subsequently demodulated during the first receive window and the frame was intended for this end-device after address and MIC (message integrity code) checks, the end-device does not open the second receive window. This class have the minimum impact on the battery lifetime of the sensor. For all of these reasons, LoRaWAN Class A will be studied in the rest of this paper.

### 5.2. LoRa Modulation Technique

#### 5.2.1. LoRa basics

The LoRa radio has different configuration parameters: the carrier frequency, the spreading factor, the bandwidth and the coding rate [9,10]. The combination of these parameters provides different energy values and transmission ranges:

- Carrier Frequency (*CF*): The CF is the center frequency used for the transmission band. For the *SX1272* transceiver, *CF* is in the range of 863 MHz to 870 MHz in Europe.
- Spreading Factor (*SF*): The *SF* is the number of chips per symbol. Its value is an integer number between 6 and 12. The greater value of *SF*, the more capability the receiver has to move away the noise from the signal. Thus, the greater value is taken, the more time is taken to send a packet.
- Bandwidth (*BW*): The *BW* represents the range of frequencies in the transmission band [16]. It can only be chosen among three options: 125 kHz, 250 kHz or 500 kHz. If a fast transmission is required, a 500 kHz value is better. However, if a long range is needed, a 125 kHz value must be configured.
- Coding Rate (*CR*): The coding rate expression is $CR=\frac{4}{4+n}$, *n* is from 1 to 4. It denotes that every four useful bits are encoded by 5, 6, 7 or 8 transmission bits. The smaller the coding rate is, the higher the time on air is to transmit data.

The nominal bit-rate (in bits per second), is obtained taking into account these parameters. The expression of the bit-rate is given in Equation (13):

(13) $$R_{bit}=SF\cdot \frac{BW}{2^{SF}}\cdot CR$$

Table 2 presents the chirp packet length based on the *SF* parameter. Modifying this parameter provides a trade-off between increasing the communication distance and decreasing the data transfer rate. Each symbol is spread through a chirp code whose length is $2^{SF}$. The receiver divides the received code into $\frac{2^{SF}}{SF}$ length blocks which can be then decoded.

#### 5.2.2. LoRa Packet Structure

**Figure 5:**
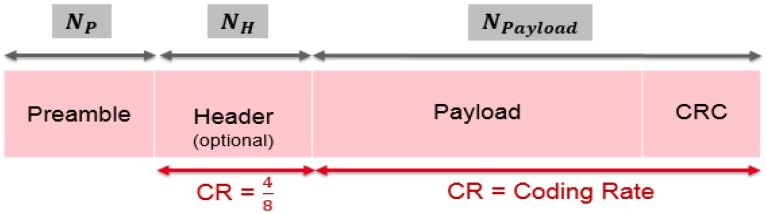
LoRa packet structure.

This part presents the LoRa frame definition. This frame starts with a preamble which is used for the synchronization between the receiver and the transmitter [9,10,11]. After the preamble, an optional header carries the size of the payload and the information about the LoRa configuration. It is noted that the header is always encoded with a $CR=\frac{4}{8}$. The payload is encoded with a variable *CR*. An optional Cyclic Redundancy Check (*CRC*) is sent at the end of the frame. Figure 5 depicts the LoRa frame content.

To calculate the time on air (or packet duration), we start with the calculation of the payload symbol [16]. For a given payload noted PL (in bytes), a spreading factor *SF* and a coding rate *CR*, the number of symbols $N_{Payload}$ used to transmit the payload can be calculated by Equation (14):

(14) $$N_{Payload}=8+max\left(ceil\left(\frac{\Theta (PL,SF)}{\Gamma (SF)}\right)\cdot \frac{1}{CR},0\right),$$

where ceil indicates the ceiling function, Θ(PL,SF)=8·PL−4·SF+16+28−20·H; with H=0 when the header is enabled and H=1 when no header is present and Γ(SF)=SF−2·DE; with DE=1 when the low data rate optimization is enabled and DE=0 for the other case. Then, the time on air is the sum of the preamble and the payload duration:

(15) $$T_{Packet}=T_{Preamble}+T_{Payload},$$

where $T_{Packet}$, $T_{Preamble}$ and $T_{Payload}$ are, respectively, the packet duration, the preamble duration and the payload duration. The preamble duration is expressed as:

(16) $$T_{Preamble}=\left(4.25+N_{P}\right)\cdot T_{Symbol},$$

where $N_{p}$ is the preamble symbol number and $T_{Symbol}$ is the symbol period. It is defined as the time taken to send $2^{SF}$ chips. Then, recalling that the bandwidth BW is equal to the chip rate, the symbol period is given by Equation (17):

(17) $$T_{Symbol}=\frac{2^{SF}}{BW}$$

The payload duration is defined in Equation (18):

(18) $$T_{Payload}=N_{Payload}\cdot T_{Symbol}$$

We introduce the energy per useful bit $E_{bit}$ in this paper, which is an important metric to evaluate the power consumption of the sensor node. The $E_{bit}$ expression is given in Equation (19):

(19) $$E_{bit}=\frac{E_{Total}}{8\cdot PL}=\frac{Pcons\left(P_{Tr}\right)\cdot T_{Packet}}{8\cdot PL},$$

where PL, $E_{Total}$ and $Pcons(P_{Tr})$ the payload size, the total consumed energy and the total consumed power which depends on transmission power. Using Equations (15), (16) and (18), the energy per useful bit can be rewritten in the following equation:

(20) $$E_{bit}=\frac{Pcons\left(P_{Tr}\right)\cdot T_{Packet}}{8\cdot PL}=\frac{Pcons\left(P_{Tr}\right)\cdot \left(N_{Payload}+N_{P}+4.25\right)\cdot T_{Symbol}}{8\cdot PL}$$

Replacing $T_{Symbol}$ with its expression in Equation (17), we can write $E_{bit}$ as a function of the spreading factor SF. As can be seen, the $E_{bit}$ is an increasing function according to SF:

(21) $$E_{bit}=\frac{Pcons\left(P_{Tr}\right)\cdot \left(N_{Payload}+N_{P}+4.25\right)\cdot 2^{SF}}{8\cdot PL\cdot BW}$$

Table 3 presents different transmission power levels and corresponding current consumption that can be used with the LoRa *SX1272* transceiver [29].

#### 5.2.3. Acknowledgment Transmission, Communication Range and Sensitivity

**Figure 6:**
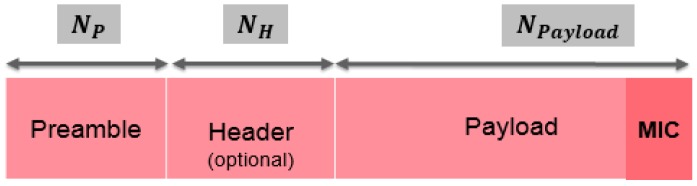
LoRa ACK structure.

As presented in Figure 6, following each uplink transmission, the sensor node opens two short receive slots in the Class A. The first RX1 message uses the same frequency channel as the uplink frame (the RX Delay 1 is equal to 1 s for the *SX1272* LoRa transceiver). The second receive window RX2 uses a fixed configurable frequency and data rate (the RX Delay 2 is equal to 2 s for the *SX1272*) [30]. These RX messages can be considered as a transmission acknowledgement (ACK). The internal structure of the ACK message is depicted in Figure 6. The ACK packet is ending with the Message Integrity Code (MIC).

To calculate the communication range of LoRaWAN system, different statements can be found reaching from multiple kilometers to a maximum range of 15 km [27]. The communication range noted *d* can be estimated using the path-loss $L_{path}$ expression:

(22) $$L_{path}={\left(\frac{4\cdot \pi \cdot f}{c}\right)}^{2}\cdot d^{n},$$

where *f* is the LoRa frequency, *c* is the light speed and *n* is the path-loss exponent, it can be equal to 2 (for free space), 3 (for urban area) and 6 (for high obstruction). Then, the link budget $L_{budget}$ for the transmission path is expressed as:

(23) $$L_{budget}=\frac{P_{Tr}}{S_{R}\left(SF,BW\right)},$$

where $P_{Tr}$ and $S_{R}\left(SF,BW\right)$ are, respectively, the transmitted power and the receiver sensitivity, which depends on spreading factor and bandwidth. The sensitivity of the receiver is defined by the minimum received power to detect the signal. This sensitivity is obtained for a minimum Signal to Noise Ratio (SNR) equal to $\frac{E_{bit}}{N_{0}}$, where $E_{bit}$ is the energy per bit and $N_{0}$ is the noise power spectral density. Define $SNR_{0}$ equal to this minimum:

(24) $$SNR_{0}=\frac{E_{bit}}{N_{0}}$$

We have $E_{bit}=S_{r}\cdot T_{bit}$ where $S_{r}$ is the received power and $T_{bit}$ is the bit duration. The relation between $T_{bit}$ and $T_{chirp}$ is $T_{bit}=T_{chirp}\cdot 2^{SF}$. Assuming that $T_{chirp}=\frac{1}{BW}$, Equation (24) can be written:

(25) $$SNR_{0}=\frac{S_{r}\cdot 2^{SF}}{NF\cdot k\cdot T\cdot BW}$$

Then, Equation (25) can be rewritten as the following:

(26) $$S_{r}=\frac{SNR_{0}\cdot N\cdot k\cdot T\cdot BW}{2^{SF}}$$

Using the datasheet of the *SX1272* transceiver [29], the receiver sensitivity can be defined in the following equation:

(27) $$S_{R}\left(SF,BW\right)=SNR\left(SF\right)\cdot N_{0}=SNR\left(SF\right)\cdot NF\cdot k\cdot T\cdot BW,$$

where *NF*, *k*, *T* and SNR(SF) are the receiver architecture noise figure, the Kelvin constant, the temperature and the Signal to Noise Ratio, respectively. Comparing Equations (26) and (27), the SNR(SF) is given in Equation (28):

(28) $$SNR\left(SF\right)=\frac{SNR_{0}}{2^{SF}},$$

where $SNR_{0}$ equals 15 dB for the *SX1272* transceiver [29].

Then, to have the estimation value of maximum communication range of LoRa link, we assume that there are no antenna gains and set the path loss $L_{path}$ equal to $L_{budget}$. Equation (29) depicts the $L_{path}$ expression:

(29) $$L_{path}=\frac{P_{Tr}}{S_{R}\left(SF,BW\right)}=\frac{P_{Tr}}{SNR(SF)\cdot NF\cdot k\cdot T\cdot BW}=\frac{P_{Tr}\cdot 2^{SF}}{SNR_{0}\cdot NF\cdot k\cdot T\cdot BW}$$

Using Equations (22), (23), and (27)–(29), the LoRaWAN range noted *d* can be estimated as follow:

(30) $$d={\left(\frac{L_{path}}{{\left(\frac{4\cdot \pi \cdot f}{c}\right)}^{2}}\right)}^{\frac{1}{n}}={\left({\left(\frac{c}{4\cdot \pi \cdot f}\right)}^{2}\cdot \frac{P_{Tr}\cdot 2^{SF}}{SNR_{0}\cdot NF\cdot k\cdot T\cdot BW}\right)}^{\frac{1}{n}}$$

The LoRaWAN range is an increasing function according to *SF* (meaning that we should use high *SF* values to reach long LoRaWAN range).

## 6. Numerical Results and Discussions

In this section, we start by presenting a use case of our energy consumption model. Then, simulation results of LoRa/LoRaWAN are presented. We finish by discussing the performance of our energy model using different LoRaWAN modes.

### 6.1. Application Case

**Figure 7:**
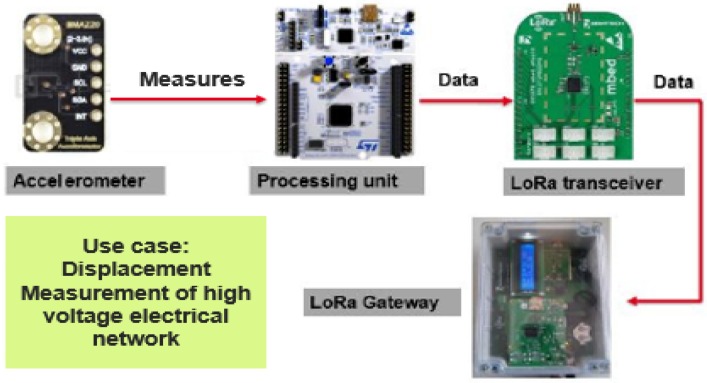
Hardware of the sensor node.

To illustrate the application of our energy model, the considered use case is the monitoring of high voltage electrical network pylons. The purpose of this application is to measure the pylon movement. The system composition is depicted in Figure 7. The whole hardware/software system is powered by a battery. The sensor unit is composed of an accelerometer which measures the pylon deviation and sends data measurements to the processing unit. The processing unit will retrieve the acceleration measurements and it will do the necessary treatment. Then, the LoRa transceiver ensures the data transmission to the corresponding gateway.

Table 4 depicts the main parameters of this application.

In summary, the communicating sensor realizes periodical measurements of the acceleration. After the sensing phase, data frames are sent to the access point using LoRa technology. Then, energy consumption of LoRa Class A will be discussed taking into account the acknowledgment transmission.

### 6.2. Modeling of LoRa/LoRaWAN

This section focuses on the LoRa modulation scheme and the LoRaWAN protocol taking into account the variation effect of the spreading factor, the coding rate and the bandwidth, which are critical parameters permitting a trade-off between energy consumption and nominal data rate [22]. Table 5 summarizes the characteristics of three LoRaWAN modes that can be used with the *SX1272* transceiver.

#### 6.2.1. Effect of *SF* and *CR* on Consumed Energy

**Figure 8:**
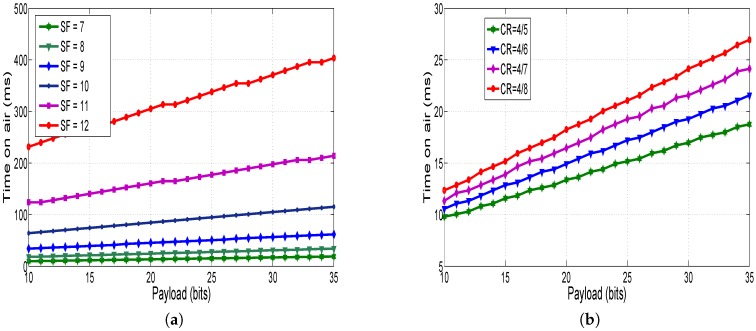
(**a**) Time on air vs. payload at different *SF*; and (**b**) time on air vs. payload at different *CR*.

Using Equation (15), Figure 8 displays the time on air as a function of payload size at different *SF* and *CR* values. The bandwidth channel is set to 500 kHz. When the *SF* is high, the time on air increases (Figure 8a), which means that the sensor node consumes more power to transmit data. The effect of *CR* on the time on air is depicted in Figure 8b. We note that increasing the number of encoding bits causes an increase of packet transmission, which allows consuming more power by the radio module.

**Figure 9:**
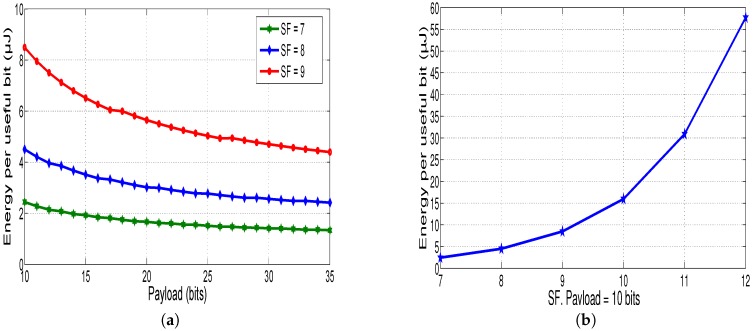
(**a**) Effect of *SF* on the consumed energy, $CR=\frac{4}{5}$; and (**b**) energy per useful bit evolution as a function of *SF*.

Figure 9a presents the consumed energy per useful bit as a function of the payload at different spreading factors (*SF*). Using Equation (21), it is noted that this energy decreases with the increase of the number of useful bits. This result is shown in Figure 9b, which depicts the evolution of the energy per useful bit as a function of *SF* for constant payload size (equal to 10 bits). As mentioned before, the greater value of *SF*, the more time is taken to send a packet, so the more consumed energy is needed to transmit data.

**Figure 10:**
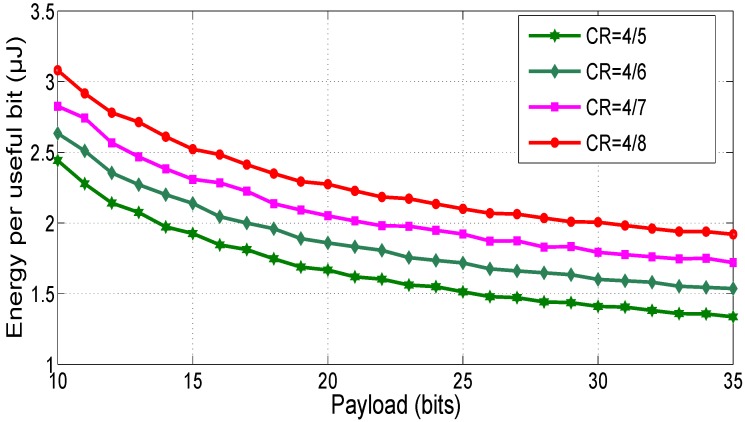
Effect of *CR* on the consumed energy, SF=7 and BW=500 KHz.

The effect of the coding rate (*CR*) on the energy per useful bit is depicted in Figure 10. When the coding rate decreases, the time on air and the consumed energy are increased.

The results presented in Figure 8, Figure 9 and Figure 10 show that optimizing the LoRa parameters such as *SF*, *CR* and payload size is a key element to reduce the consumed energy by the sensor node.

#### 6.2.2. LoRaWAN Communication Range

**Figure 11:**
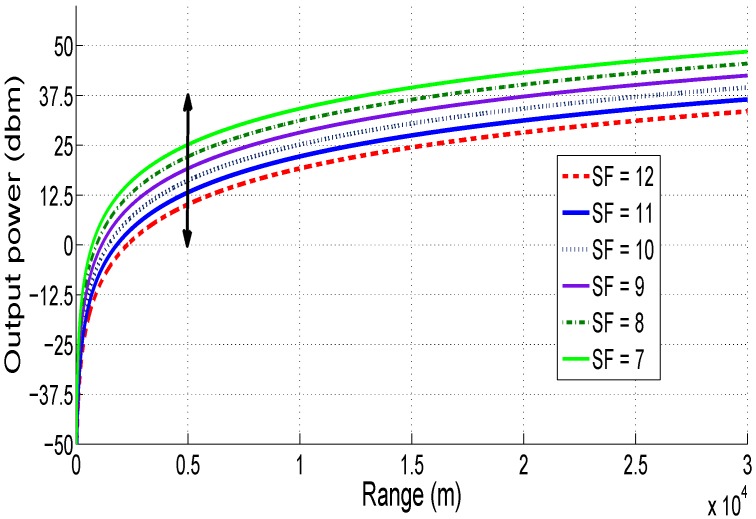
Required output power vs. LoRaWAN range.

Referring to Equation (30), Figure 11 presents the required output power as a function of the LoRaWAN communication range at different spreading factor *SF* (assuming a path-loss exponent equal to 3 for urban area). Using LoRaWAN protocol, the theoretical maximum range that can be achieved at determined power level is obtained with *SF* equal to 12. In fact, with *SF* = 12, the sensor node needs 10 dBm to transmit data for 5 km in urban area with little obstructions. However, the sensor needs 25 dBm to transmit the same data for the same distance with *SF* = 7.

**Figure 12:**
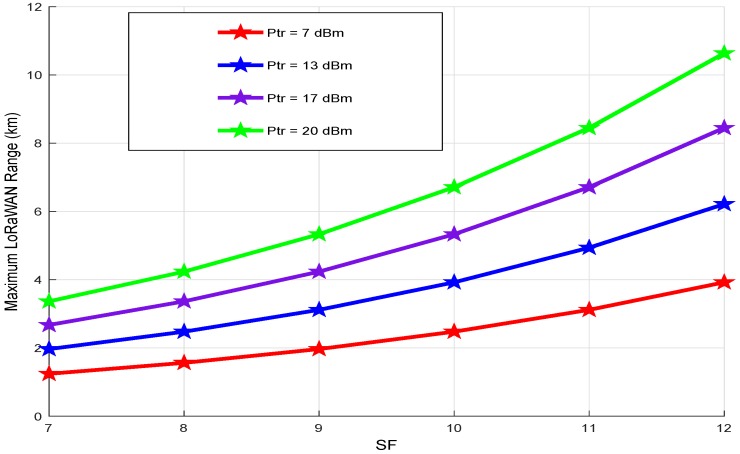
Maximum LoRaWAN range vs. *SF*.

The maximum LoRaWAN range as a function of *SF* at different transmission powers is depicted in Figure 12 using Equation (30). As we can see, if the *SF* increases, the LoRaWAN range increases. For a constant *SF* value, the LoRaWAN range increases with increasing transmission power. In fact, to reach a communication range of 4 km with *SF* equal to 9, we can use both 17 and 20 dBm. However, for high communication ranges (greater than 10 km), the transmission power must be fixed 20 dBm with *SF* equals 12. These observations are very important for the conception of our energy model: by setting a LoRaWAN transmission distance and using results in Figure 11 and Figure 12, we can have an idea about the optimal output power and spreading factor to use for our application, which help to minimize the energy consumption by the sensor node.

**Figure 13:**
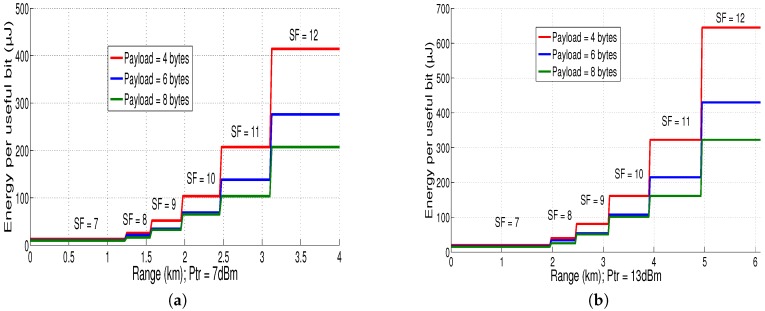
(**a**) $E_{bit}$ vs. LoRaWAN range at different payloads ($P_{Tr}$ = 7 dBm); and (**b**) $E_{bit}$ vs. LoRaWAN range at different payloads ($P_{Tr}$ = 13 dBm).

Using Equations (21) and (30), Figure 13 presents the energy per useful bit evolution as a function of maximum range at different payload sizes for both 7 dBm and 13 dBm. The maximum range is reached with *SF* equals to 12 for both cases (we can reach 4 km with 7 dBm in Figure 13a and 6.1 km with 13 dBm in Figure 13b). It can be noticed that, if the payload size increases, the energy per useful bit decreases for high *SF* values (for low *SF* values, the payload variation does not have important effect on energy per useful bit). To reach 3 km with payload size equal to 4 bytes, we must configure *SF* at 11 with transmission power 7 dBm which consumes 0.21 mJ/bit. However, using transmission power 13 dBm, we can use *SF* equals 9 to reach the same distance 3 km with energy consumption of only 0.08 mJ/bit.

In summary, using results found in Figure 11, Figure 12 and Figure 13, we note that there is a trade-off among the LoRaWAN communication range, the spreading factor and the transmission power. In this case, the increase of the transmission power is more interesting in terms of consumed energy than the increase of the spreading factor. Then, we can refer to these results to find the best LoRaWAN parameter values for our application and to optimize the energy consumption of the sensor node.

### 6.3. Full Communicating Sensor-Energy Consumption Results

In this part, we will evaluate the performance of our energy consumption model using LoRaWAN mode 3 (Table 5) because the provided range is enough for our application and this allows saving the use of the battery. For that, the following scenario is proposed:

The sensor node realizes measurements of the acceleration and transmits acceleration value every 30 s. It is noted that the microcontroller operating frequency is equal to 4 MHz in this study. Table 6 shows the power and time parameters of the model. These parameters are given in the *BMA220*, *STM32L073* and *SX1272* datasheets [29,32,33].

**Figure 14:**
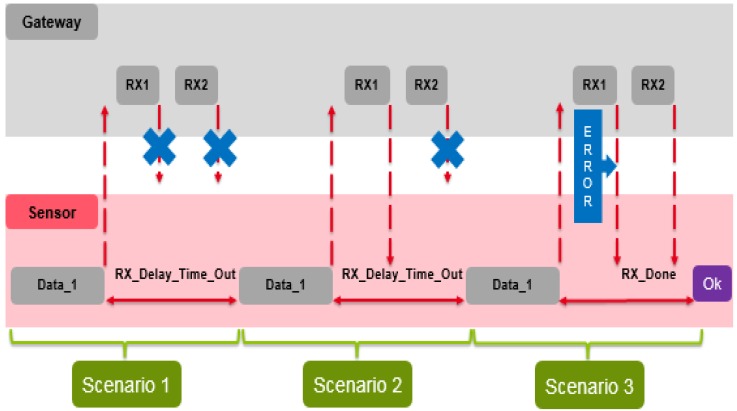
Proposed LoRaWAN (Class A) scenarios.

Figure 14 depicts possible scenarios of the sensor node based on LoRaWAN Class A. The first scenario is to transmit data to the gateway without receiving both RX1 and RX2. The second one is to transmit data and receive RX1 without receiving RX2. The third scenario is to transmit data and demodulate RX1 which contains transmission error, so that the node must demodulate RX2.

#### 6.3.1. Consumed Energy: Scenario 1

**Figure 15:**
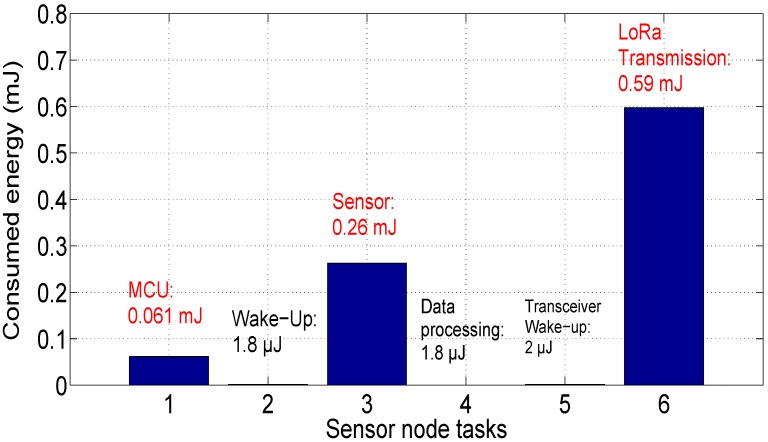
Energy consumption of sensor node: Scenario 1.

Figure 15 presents the power consumption amount by each task of the communicating sensor. As indicated before, the main energy consumers are the microcontroller unit ($E_{MCU}$ = 0.061 mJ), the sensor unit ($E_{m}$ = 0.26 mJ) and the RF unit ($E_{Tr}$ = 0.59 mJ). It is noted that the transceiver part is the main energy consumer in the sensor node. In this case, the LoRa frame must be retransmitted by the sensor node. As indicated in [34], if the downlink message is lost for any reason, the LoRa specifications recommends to transmit packet up to eight times.

**Figure 16:**
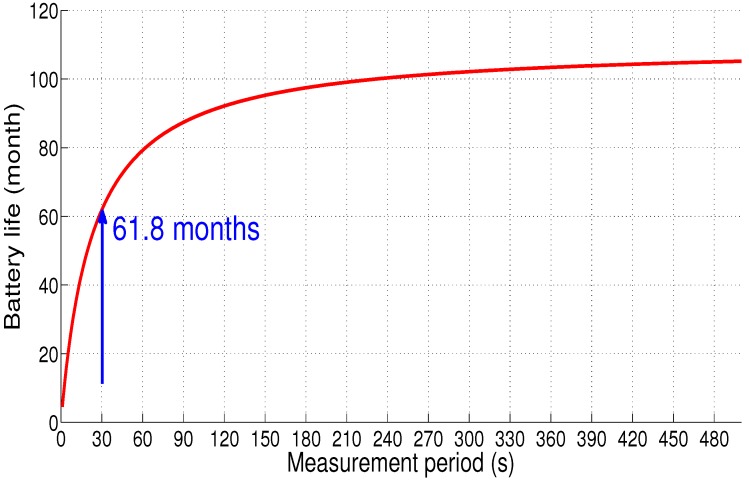
Sensor node autonomy: Scenario 1.

Figure 16 depicts the battery life as a function of measurement period, with a capacity of 950 mAh and a supply voltage of 3.3 V, the sensor node autonomy is estimated at 5 years, 1 month and 24 days (61.8 months) when the measurement period is equal to 30 s.

#### 6.3.2. Consumed Energy: Scenario 2

**Figure 17:**
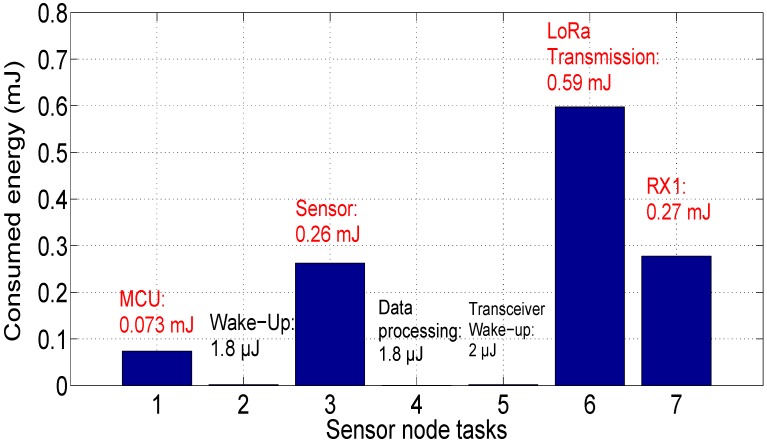
Energy consumption of sensor node: Scenario 2.

In this scenario, we suppose that the sensor node transmits data to the gateway, then it receives the first acknowledgment RX1 to confirm data transmission (in this case, the sensor node will not demodulate the second acknowledgment RX2). The energy consumption by the communicating sensor is given in Figure 17. As can be seen, the difference from Scenario 1 are the dissipated energy by the LoRa receiver ($E_{R}$ = 0.27 mJ) and the consumed energy by the MCU unit.

**Figure 18:**
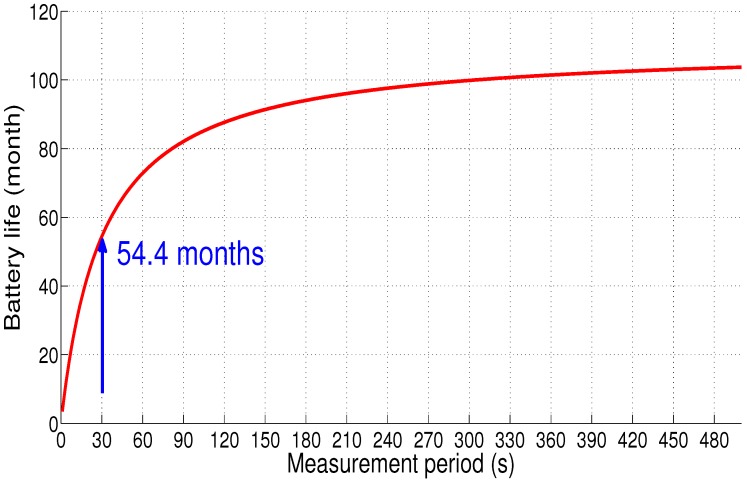
Sensor node autonomy: Scenario 2.

Figure 18 presents the sensor node lifetime using the same battery characteristics (capacity equals 950 mAh and supply voltage of 3.3 V). The sensor node autonomy is estimated at 4 years, 6 months and 12 days (54.4 months) for the same measurement period (30 s), which is 7.4 months shorter than Scenario 1 (i.e., there is a loss equal to 12% of the battery life compared to Scenario 1).

#### 6.3.3. Consumed Energy: Scenario 3

**Figure 19:**
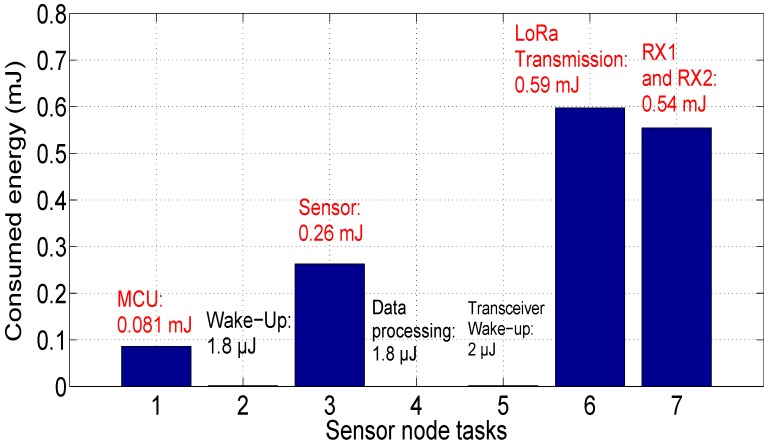
Energy consumption of sensor node: Scenario 3.

For this scenario, the communicating sensor transmits data to the gateway and receives the RX1 acknowledgment which contains transmission error for example. The sensor node must demodulate the RX2 acknowledgment to verify the transmission success (which means that it will consume more energy than Scenario 2). The dissipated energy by the communicating sensor is given in Figure 19. We remark that the consumed energy is tdouble that consumed by the LoRa receiver ($E_{R}$ = 0.54 mJ).

**Figure 20:**
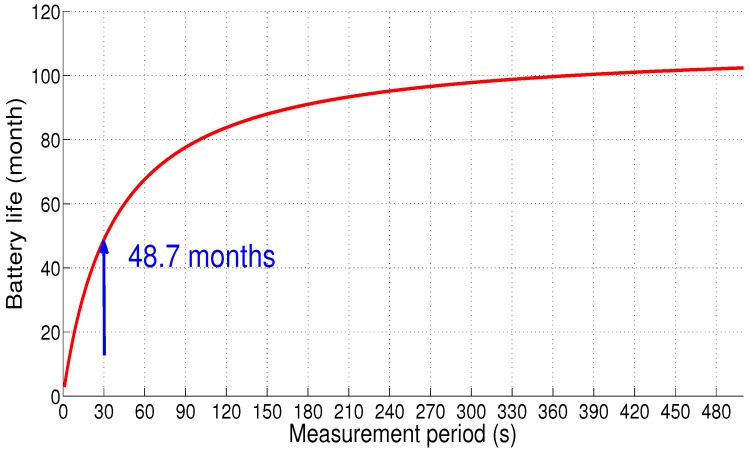
Sensor node autonomy: Scenario 3.

The sensor node lifetime is depicted in Figure 20. The autonomy of the node is estimated at 4 years and 21 days (48.7 months) for this case (i.e, there is a loss equal to 22.2% of the battery life compared to Scenario 1).

#### 6.3.4. Comparison between Proposed Scenarios

Table 7 presents comparison between the proposed scenarios. As we can see, the sensor node lifetime in Scenario 1 is higher than Scenarios 2 and 3. These results show the energy consumption cost of receiving downlink messages from the gateway.

**Figure 21:**
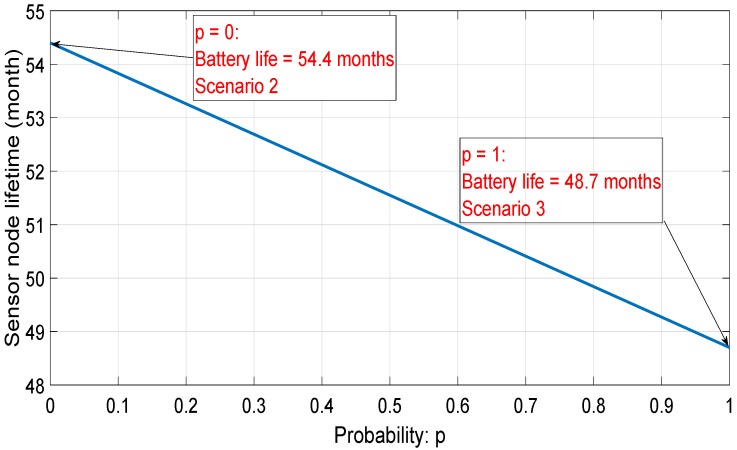
Battery life vs. probability of having Scenario 3.

Equation (31) depicts the total consumed energy $E_{total}$ as a function of the probability of having Scenario 3 noted *p*:

(31) $$E_{total}=\left(1-p\right).E_{scenario2}+p.E_{scenario3},$$

where $E_{scenario2}$ and $E_{scenario3}$ are the total consumed energies using Scenarios 2 and 3, respectively. Using results in Section 6.3.2 and Section 6.3.3, the sensor node autonomy as a function of probability *p* is given in Figure 21. We remark that the sensor node lifetime decreases from 54.4 months when *p* = 0 (which is the sensor node lifetime using Scenario 2) to 48.7 months when *p* =1 (the probability that Scenario 3 occurs).

In the ideal case (data transmission with acknowledgment reception and without transmission error), Scenario 2 is the most frequent one (meaning that the probability *p* is closed to 0). Thus, Scenario 2 is selected for the rest of this paper.

### 6.4. Effect of LoRaWAN Mode in Sensor Node Lifetime

Table 8 shows the effect of different LoRaWAN modes on the sensor node autonomy. As mentioned in Table 5, Mode 3 is the higher data rate with lower range which have the minimum battery impact (the sensor node autonomy can reach 4 years, 6 months and 12 days). However, Mode 1 gives the shortest battery life because of the high value of *SF* (the sensor node consumes a lot in this case).

### 6.5. Effect of Microcontroller Frequency in Sensor Node Lifetime

**Figure 22:**
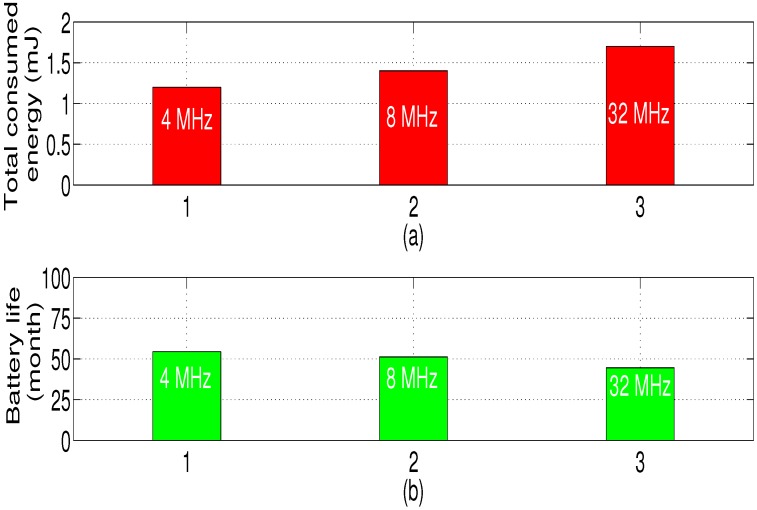
Total energy consumption for different microcontroller frequencies (**a**), Battery life (**b**) (Mode 3, Scenario 2).

To show the effect of the microcontroller operating frequency $f_{MCU}$ on the sensor node autonomy, we refer to Figure 22 (the voltage supply is set to 3.3 V) [33]. We note that the energy consumption of the node depends on the microcontroller speed (it increases with frequency). In fact, the higher the microcontroller frequency is, the lower the processing time is (meaning that the processing time $T_{proc}\left(f_{MCU}\right)$ decreases) which causes the decrease of the microcontroller time duration $T_{MCU}\left(f_{MCU}\right)$, but the higher the amount of the microcontroller power $P_{ON}\left(f_{MCU}\right)$ is. Consequently, the node autonomy decreases with increasing the MCU frequency.

## 7. Conclusions

Energy consumption is one of the most constraining requirements for design and implementation of communicating sensors. In this paper, we present an optimized energy model for sensor nodes using LoRa/LoRaWAN technologies. This model allows the analysis of different LoRaWAN modes and scenarios for a specific Internet of Things application based on LoRaWAN Class A. In fact, to evaluate the energy consumption of the sensor node, we proposed different LoRaWAN scenarios. We concluded that receiving a transmission acknowledgment consumes an energy amount which reduces the sensor node lifetime. Then, the proposed energy model was evaluated using different LoRaWAN modes: this mode must be optimized to minimize the dissipated energy by the communicating sensor.

The developed model also allows studying the impact of the hardware and the software choices into the node autonomy. We showed through numerical results that the consumed energy changes with different LoRa/LoRaWAN parameters such as spreading factor, coding rate, payload size and bandwidth. Optimizing these parameters is very important to reduce the energy consumption of the sensor node.

The microcontroller operating frequency plays important role in optimizing the sensor node lifetime. Increasing the microcontroller frequency has an effect on the consumed energy which reduces the sensor node autonomy.

Besides, we showed through the optimization study that there is a trade-off between the LoRaWAN communication range, the spreading factor and the transmission power. This optimization study is very interesting to choose and configure LoRa/LoRaWAN parameters. In fact, the increase of transmission power is more interesting in terms of consumed energy per useful bit than the increase of spreading factor.

Finally, to show the real application case of our energy model, we considered a connected sensor for IoT applications. A specific application is discussed in this paper which is the monitoring of high voltage electrical network pylons. Using the proposed energy model, the energy consumption of LoRaWAN Class A and the sensor node lifetime can be estimated taking into account an acknowledgment transmission.

In future works, this energy model could be used in power management algorithms for communicating sensor powered by energy harvesting sources in order to maximize the sensor node lifetime.

## Figures and Tables

**Table 1:** LoRaWAN class comparison.

Class	Brief Description	Power Consumption
Class A	Sensor initiate communication, downlink after transmission	Most energy efficient
Class B	Slotted communication synchronized with beacon frames	Efficient with controlled downlink
Class C	Devices listen continuously, downlink without latency.	high power consumption

**Table 2:** Chirp code length.

Spreading Factor: *SF*	Chirp Code Length (Bit)
7	128
8	256
9	512
10	1024
11	2048
12	4096

**Table 3:** LoRa *SX1272* characteristics.

Transmission Power (dBm)	Power Consumption (mW)
20	412.5
17	297
13	92.4
7	95.4

**Table 4:** Parameters of the application.

Parameters	Values
Useful bits to transmit	32 bits
Battery capacity	950 mAh
Self-discharge current of the battery	$7.5\times 10^{-3}$ mA
Transmission power	13 dBm
Supply voltage (MCU, transceiver)	3.3 V
Supply voltage (Sensor unit)	2 V

**Table 5:** Different LoRaWAN modes [31].

Modes	Characteristics	Explanations
Mode 1	BW=125 kHz, SF=12, $CR=\frac{4}{5}$	Largest distance mode (max range and slow data rate)
Mode 2	BW=250 kHz, SF=10, $CR=\frac{4}{5}$	Intermediate mode
Mode 3	BW=500 kHz, SF=7, $CR=\frac{4}{5}$	Minimum range, high data rate and minimum battery impact

**Table 6:** Characteristics of sensor node tasks.

Task	Time Duration (ms)	Consumed Power (mW)
Sensor (*BMA220*)	25	10.5
Data transmission (*SX1272*)	6.5	92.4
MCU *STM32L073* (4 MHz)	33.5	1.8

**Table 7:** Comparison between different scenarios.

Scenario	Characteristics	RF Energy Consumption (mJ)	Sensor Autonomy (Months)
Scenario 1	TX; RX1 and RX2 not done	$E_{Tr}$ = 0.59; $E_{R}$ = 0	61.8
Scenario 2	TX; RX1 done; RX2 not done	$E_{Tr}$ = 0.59; $E_{R}$ = 0.27	54.4
Scenario 3	TX; RX1 not done; RX2 done	$E_{Tr}$ = 0.59; $E_{R}$ = 0.54	48.7

**Table 8:** Comparison between different LoRaWAN modes (using Scenario 2).

Mode	Total Consumed Energy Per Transmission (mJ)	Sensor Node Autonomy (Month)
Mode 1	115	1.12
Mode 2	14.7	8.2
Mode 3	1.2	54.4
